# Streptococcus agalactiae Infective Endocarditis in a Young Immunocompetent Male

**DOI:** 10.7759/cureus.55247

**Published:** 2024-02-29

**Authors:** Pradeep Kumar Mada, Muhammad H Khan, Timothy Trotter

**Affiliations:** 1 Infectious Diseases, Comanche County Memorial Hospital, Lawton, USA; 2 College of Osteopathic Medicine, Michigan State University, East Lansing, USA; 3 Cardiothoracic Surgery, Comanche County Memorial Hospital, Lawton, USA

**Keywords:** mechanical bi-leaflet prosthesis, bilateral cerebellar infarcts, thromboembolic stroke, infective endocarditis, group b streptococcus agalactiae bacteremia

## Abstract

Group B Streptococcus endocarditis is a rare but serious condition, characterized by the infection of heart valves and associated with a high mortality rate. The emergence of antibiotic-resistant strains adds complexity to therapeutic strategies, emphasizing the importance of tailored antibiotic regimens and surgical interventions when indicated. Early diagnosis and multidisciplinary care are essential in improving patient outcomes. A 22-year-old male patient with no comorbidities was admitted with a thromboembolic stroke. MRI brain showed bilateral cerebral and cerebellar multifocal acute nonhemorrhagic infarcts. He was found to have *Streptococcus agalactiae* bacteremia, and infective mitral valve endocarditis. He underwent mitral valve replacement and IV antibiotic treatment with a successful outcome.

## Introduction

Infective endocarditis (IE) is defined as a bacterial, viral, or fungal infection of the endocardial surfaces of the heart, may include one or more cardiac valves, and can be complicated by severe valvular insufficiency, congestive heart failure, myocardial abscess, infected emboli, and a variety of immunological phenomenon. *Streptococcus (S.) agalactiae* endocarditis is rare, as native valve infective endocarditis is mostly caused by *Staphylococcus aureus *(35-40%), *Streptococcus viridians* (20%), *Staphylococcus epidermidis *(<15%), and enterococci (10%) [1]. *Streptococcus agalactiae*, also known as group B streptococcus, is a gram-positive organism that normally colonizes human genital and gastrointestinal tracts. While *S. agalactiae* is typically associated with neonatal infections, skin and soft tissue infections, and invasive diseases in the elderly, it can also be a causative agent of infectious endocarditis associated with a high mortality rate [2,3].

## Case presentation

**Figure 1:**
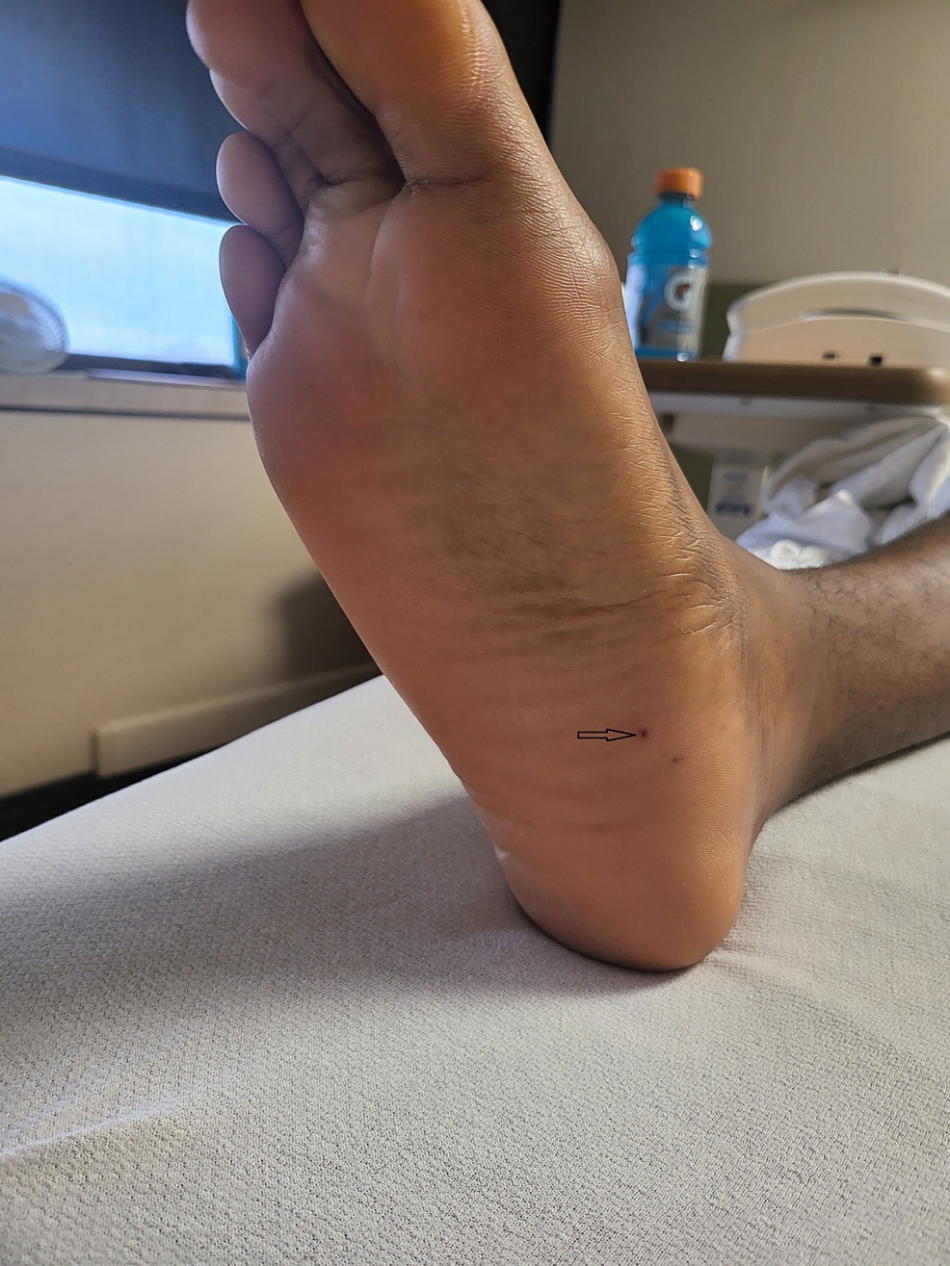
A non-tender red macule on the right sole (Black arrow)

A 22-year-old African American male with no comorbidities presented with fever and altered mental status of one-day duration. He reported chest congestion with myalgias and headache for one week, retrosternal, dull, and non-radiating chest discomfort, confusion, and fever for one day prior to admission. He had a history of significant dental procedures including multiple root canals and cavities filled in the past. He denied a history of cigarette smoking, alcohol consumption, intravenous drug use, and international travel. The patient was febrile with a temperature of 103^o^F and was hemodynamically stable on arrival. On physical exam, lungs were clear to auscultation, with normal first and second heart sounds with no added sound/murmur, and a non-tender red macule was found on the right sole (Figure 1).

**Figure 2:**
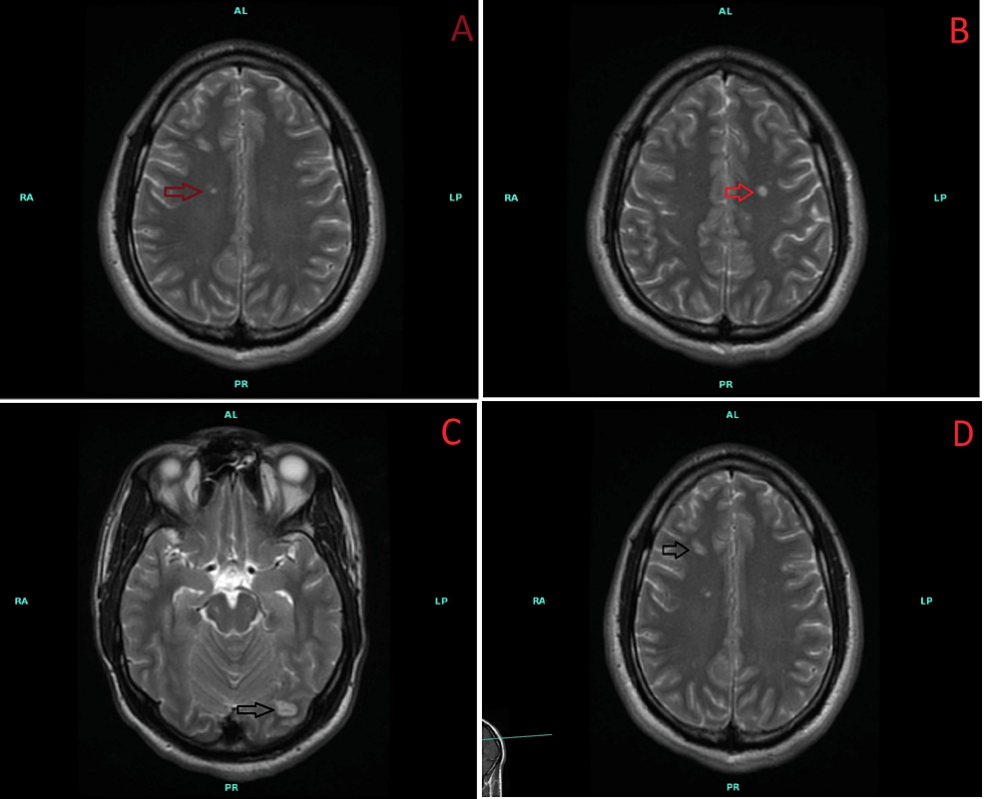
MRI brain findings showing bilateral cerebral multifocal acute non-hemorrhagic infarcts (red arrows in A and B) The largest on the left measures 4.0 x 1.8 cm on the occipital lobe subcortical white matter (black arrow in C). The largest on the right measures 1.0 x 0.5 cm on the frontal lobe subcortical white matter (black arrow in D).

Labs showed a white blood cell count of 13,650/millimeter^3 ^(mm^3^), hemoglobin of 14.9 gram/deciliter, platelets of 142,000/mm^3^, erythrocyte sedimentation rate of 35 millimeter per hour, C-reactive protein of 162.86 milligram/liter. Initial electrocardiogram showed sinus rhythm, heart rate of 91 beats per minute, and no T wave abnormalities or ST segment changes. A computed tomography scan of the head without contrast showed no acute intracranial process or mass lesion. Magnetic resonance imaging (MRI) brain without contrast showed bilateral cerebral and cerebellar multifocal acute non-hemorrhagic infarcts. The largest on the left measured 4.0 x 1.8 centimeter (cm) on the occipital lobe subcortical white matter. The largest on the right measured 1.0 x 0.5 cm on the frontal lobe subcortical white matter. Bilateral cerebellar infarcts were sub-5 millimeter (mm) in size concerning for thromboembolic stroke (Figure 2).

Blood cultures grew *Streptococcus agalactiae* in two sets. Two-dimensional (2-D) echocardiography revealed normal left ventricular systolic function, left ventricular ejection fraction (LVEF) of 65%, and 1.51x1.33 cm pedunculated and mobile echo density on the atrial aspect of the anterior wall leaflet of the mitral valve, most likely vegetation, with no evidence of mitral stenosis, but trace-mild regurgitation was recorded (Video 1).

**Video 1:** 2D echo findings showing a 1.51x1.33 cm pedunculated and mobile echo density on the atrial aspect of the anterior wall leaflet of the mitral valve, most likely a vegetation

**Figure 3:**
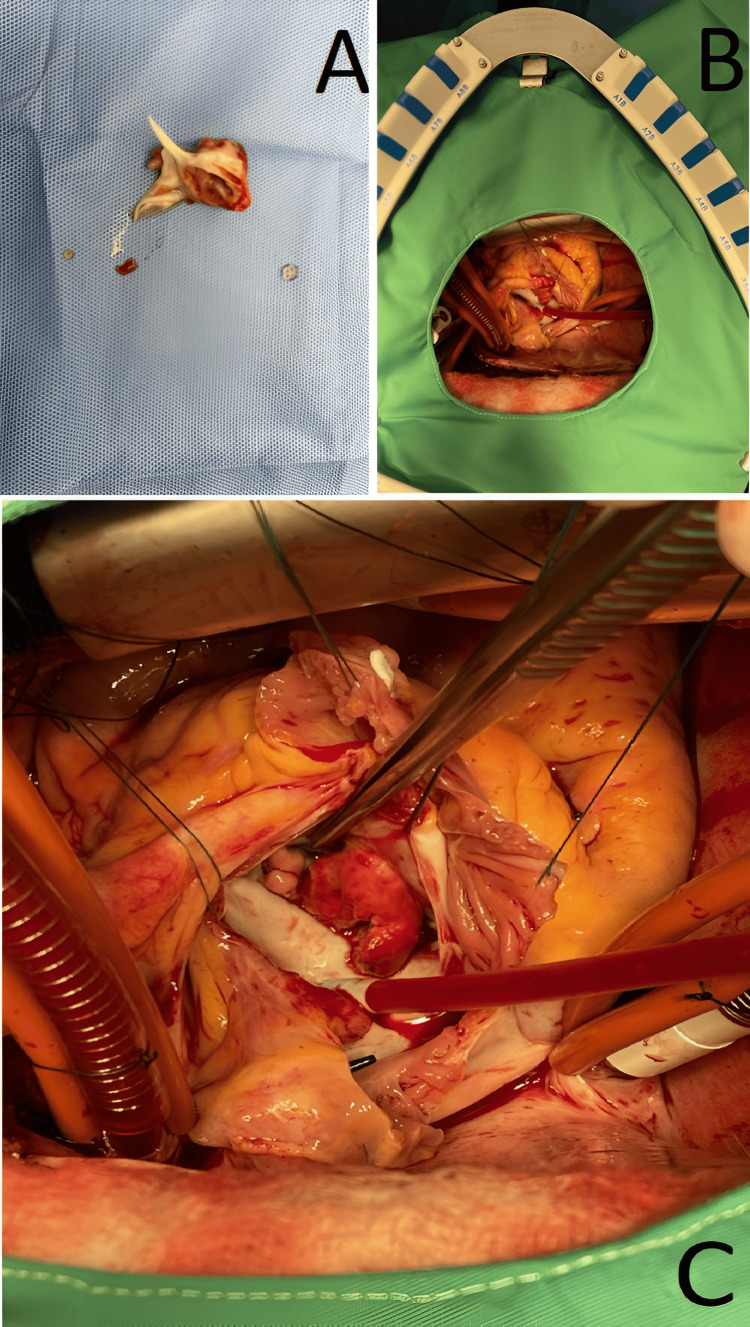
Surgical findings A. The surgically resected large vegetation attached to the anterior leaflet of the mitral valve; B. The vegetation prior to resection of the mitral leaflets. The patient's head is to the left side of the image and the superior vena caval cannula is present on the left side of the photograph. Using a Guiraudon trans atrial septal incision, which opens the left atrium through the interatrial septum, the mitral valve is exposed, demonstrating the extensive reddish-colored vegetation protruding and essentially replacing the anterior leaflet of the mitral valve; C. The patient's head is to the left of the photograph. The large, reddish-colored vegetation is seen to be protruding from the mitral annulus and replacing the anterior leaflet. The tip of the other forceps seen in the photograph is placed between vegetations protruding from the mitral valve at the level of the anterolateral commissure.

He underwent mitral valve replacement with a 33 mm On-X mechanical bi-leaflet prosthesis (the valve orifice was 25 mm) and debridement of anterior leaflet mitral valve endocarditis (Figure 3).

Mitral valve tissue cultures were also positive for *Streptococcus agalactiae* (Group B). The histology of the mitral valve tissue revealed active endocarditis with vegetation that demonstrated microorganisms (bacteria) with routine hematoxylin and eosin stain. He was treated with intravenous (IV) penicillin G 4 million units every 4 hours and IV gentamicin 1 mg/kg every 8 hours for 2 weeks followed by IV ceftriaxone 2 gram every 12 hours for 4 more weeks (Table 1) with successful outcome. Follow-up 2D echo showed mild concentric left ventricular hypertrophy, with an EF of 55 to 60%. The mechanical mitral valve was visualized with no evidence of stenosis or regurgitation. 

**Table 1:** Streptococcus agalactiae sensitivity report

Antibiotic	Streptococcus agalactiae (Group B) minimum inhibitory concentration (MIC)	
Ampicillin (AM)	<=0.06	Sensitive
Clindamycin (CD)	>0.5	Resistant
Erythromycin (E)	<=0.06	Sensitive
Levofloxacin (LVX)	1	Sensitive
Penicillin (P)	<=0.03	Sensitive
Ceftriaxone (CAX)	<=0.25	Sensitive

## Discussion

Infective endocarditis (IE) has serious complications, including stroke, which was seen in our patient [3]. While the pathogenesis of IE and emboli formation is not completely known, it is thought that a fibrin-platelet clot forms after endothelial injury, followed by the adherence of microorganisms like *S. agalactiae* to the clot, forming vegetations [4]. Particles from the vegetation can then disseminate, forming emboli, which have the ability to occlude vessels and damage critical organs [4]. In a case series of infective endocarditis secondary to *S. agalactiae*, the authors described that only a few (4 patients; 13%) presented with stroke and all were older than 50 years [5]. *S. agalactiae* endocarditis is an aggressive disease with a high rate of valvular and systemic complications. Early cardiac intervention is typically required because of heart failure and emboli. In that study, 40% of patients underwent cardiac surgery because of extensive valve destruction, and the incidence of emboli was also very high (50%) [5]. Other case reports on this topic involved patients with comorbidities such as diabetes mellitus, hypertension, alcohol use disorder, valvular disease, or other predisposing conditions [6-8]. This contrasts with our patient, a healthy young man who presented with thromboembolic stroke, making this case especially unique due to the combination of the causative organism, the patient's age, symptoms, and presentation. Though our patient had a significant history of dental procedures in the past, the data suggest that dental procedures do not significantly contribute to the risk of IE, arguing against the rationale behind the use of antibiotic prophylaxis for dental procedures except for patients with underlying cardiac conditions associated with the highest risk of adverse outcome from infective endocarditis [9].

The current management of infective endocarditis caused by *S. agalactiae* encompasses both medical and surgical interventions. The European Society of Cardiology recommends the administration of penicillin G, amoxicillin, or ceftriaxone for four weeks in patients with native valve endocarditis or six weeks in patients with prosthetic valve endocarditis. The addition of gentamicin for two weeks is recommended in both scenarios [10]. The indications for surgery include severe heart failure, severe valve dysfunction, prosthetic valve infection, invasion beyond the valve leaflets, recurrent systemic embolization, and large mobile vegetation >10 mm. In cases of embolic stroke, the decision becomes more complex due to the potential transformation to hemorrhagic stroke associated with anticoagulant therapy prior to surgery [11]. Interestingly, in a study looking at postoperative stroke incidence, no significant difference was found between patients who did and did not have microhemorrhage, suggesting that transformation to hemorrhagic stroke is not a contraindication to surgery [12]. If surgery is indicated, timing is paramount, as it can significantly impact the patient outcome, influencing the risk of mortality and systemic embolic events [13].

This case report highlights the importance of considering atypical presentations and etiologies of infective endocarditis. While *S. agalactiae* infective endocarditis is rare, physicians should keep their differentials broad in similar cases.

## Conclusions

*S. agalactiae* is an infrequent cause of infective endocarditis with a high mortality rate, although the prognosis of native valve infective endocarditis has improved in recent years, likely due to increased early cardiac surgical interventions. Given the potential for complications and the emergence of antibiotic resistance, a multidisciplinary approach involving infectious disease specialists, cardiologists, and surgeons is crucial for improving patient outcomes. The emergence of antibiotic-resistant strains of *S. agalactiae* is a growing concern. This necessitates ongoing surveillance and monitoring of susceptibility patterns to ensure that treatment regimens remain effective.
